# Modified Maturity Offset Prediction Equations: Validation in Independent Longitudinal Samples of Boys and Girls

**DOI:** 10.1007/s40279-017-0750-y

**Published:** 2017-06-12

**Authors:** Sławomir M. Kozieł, Robert M. Malina

**Affiliations:** 1 Department of Anthropology, Hirszfeld Institute of Immunology and Experimental Therapy, Polish Academy of Sciences, Wrocław, Poland; 2 Department of Kinesiology and Health Education, University of Texas, Austin, TX USA; 3 10735 FM 2668, Bay City, TX 77414 USA

## Abstract

**Background:**

Predicted maturity offset and age at peak height velocity are increasingly used with youth athletes, although validation studies of the equations indicated major limitations. The equations have since been modified and simplified.

**Objective:**

The objective of this study was to validate the new maturity offset prediction equations in independent longitudinal samples of boys and girls.

**Methods:**

Two new equations for boys with chronological age and sitting height and chronological age and stature as predictors, and one equation for girls with chronological age and stature as predictors were evaluated in serial data from the Wrocław Growth Study, 193 boys (aged 8–18 years) and 198 girls (aged 8–16 years). Observed age at peak height velocity for each youth was estimated with the Preece–Baines Model 1. The original prediction equations were included for comparison. Predicted age at peak height velocity was the difference between chronological age at prediction and maturity offset.

**Results:**

Predicted ages at peak height velocity with the new equations approximated observed ages at peak height velocity in average maturing boys near the time of peak height velocity; a corresponding window for average maturing girls was not apparent. Compared with observed age at peak height velocity, predicted ages at peak height velocity with the new and original equations were consistently later in early maturing youth and earlier in late maturing youth of both sexes. Predicted ages at peak height velocity with the new equations had reduced variation compared with the original equations and especially observed ages at peak height velocity. Intra-individual variation in predicted ages at peak height velocity with all equations was considerable.

**Conclusion:**

The new equations are useful for average maturing boys close to the time of peak height velocity; there does not appear to be a clear window for average maturing girls. The new and original equations have major limitations with early and late maturing boys and girls.

**Electronic supplementary material:**

The online version of this article (doi:10.1007/s40279-017-0750-y) contains supplementary material, which is available to authorized users.

## Key Points

**Table:** 

Predicted maturity offset and age at peak height velocity (PHV) were dependent upon chronological age and probably size at prediction; predicted offset systematically decreased and age at PHV systematically increased with chronological age at prediction.
Variation in predicted ages at PHV with the new equations was systematically reduced compared with predictions with the original equations and observed ages at PHV.
Predicted age at PHV with the new equations was quite accurate for average maturing boys within ±1 year of observed PHV; a corresponding window for average maturing girls was not apparent.
Predicted ages at PHV were systematically later than observed ages at PHV in early maturing boys and girls, and systematically earlier than observed ages at PHV in late maturing boys and girls.
The Bland–Altman regressions indicated a lack of fit between predicted and observed ages at PHV within each chronological age group and within each year before and after PHV; slopes for the new equations were greater than those for the original equations.
Intra-individual variation in predicted ages at PHV with the new and original equations was considerable.

## Introduction

Individual differences in biological maturation are important considerations in youth sport and talent development, and in studies of physical activity [1]. Established indicators of maturity status [skeletal age (SA), secondary sex characteristics] are often perceived as impractical and invasive. As such, there is interest in ‘non-invasive’ indicators [1, 2]. Predicted maturity offset, defined as the time before or after peak height velocity (PHV), is one such indicator; chronological age (CA) at prediction minus offset provides an estimate of age at PHV [3].

Both predicted offset and age at PHV are increasingly used in studies of sport, performance and physical activity among youth spanning late childhood through adolescence [1]. The interval of PHV is also central to the long-term athlete development model [4, 5], while attention to individual differences in biological maturation is increasingly recommended in the design of conditioning and training programmes for youth [6–8] and in talent development [9].

Although the original sex-specific prediction equations [3] for the prediction of maturity offset are widely used, validation studies indicated several limitations in both sexes [10–12]. The original equations have since been refined, simplified and modified to include fewer predictors and to accommodate the lack of a measurement of sitting height in many studies [13]. Published applications of the new equations are limited to date [14–17].

The purpose of this study is to validate the new maturity offset prediction equations. It specifically compares predicted ages at PHV based on the new equations with observed age at PHV in longitudinal samples of boys and girls used in validations of the original equations [10, 11]. Predictions based on the original equations [3] are included for comparison. Predictions are compared by CA group from late childhood through adolescence and by years before and after observed age at PHV. Comparisons are also made among boys and girls of contrasting maturity status (early, average, late) defined by observed age at PHV.

## Methods

### Participants

Data are from the Wrocław Growth Study, which followed Polish boys and girls from 1961 through 1972. The study was approved and funded by the Polish Academy of Sciences. Details of the study have been summarised [10, 11]. The analysis is based on serial data for 193 boys aged 8–18 years and 199 girls aged 8–16 years. The samples represented 45% of the total number measured in 1961 and did not differ in height at 8 years from those who dropped out. Based on paternal education and occupation, families of the children were characteristic of the Wroclaw population. Polish law at the time did not require written consent of parents for children to participate in growth studies. Parental presence at annual examinations was accepted as implicit informed consent. However, children were free to withdraw at any time.

### Anthropometry

Body mass, stature and sitting height were measured annually by trained and experienced staff in the laboratory of the Institute of Anthropology; the protocols and estimated measurement variability have been reported [10, 11]. Leg length was estimated as stature minus sitting height.

### Age at Peak Height Velocity

Serial heights for each individual were fitted with the Preece–Baines Model 1 to estimate age at PHV [10, 11]. Descriptive statistics were as follows: girls, 11.89 ± 1.00 years, range 9.03–14.82 years; boys, 14.06 ± 1.11 years, range 11.45–7.34 years.

### Predicted Maturity Offset

Two new sex-specific equations (labelled Moore-1) were recommended [13]:

$${\text{Girls:}}\quad {\text{Maturity}}\;{\text{offset}}\;({\text{years}}) = - 7. 7 0 9 1 3 3+ ( 0. 0 0 4 2 2 3 2\times ( {\text{age}} \times {\text{stature)}}) ,$$

$${\text{Boys:}}\quad {\text{Maturity}}\;{\text{offset}}\;({\text{years}}) = - 8. 1 2 8 7 4 1+ (0.00 70 3 4 6 \times ({\text{age}} \times {\text{sitting}}\;{\text{height}})).$$

An alternative equation (labelled Moore-2) was also provided for boys:

$${\text{Maturity}}\;{\text{offset}}\;({\text{years}}) = - 7. 9 9 9 9 9 4 + (0.00 3 6 1 2 4 \times ({\text{age}} \times {\text{stature}})).$$

Standard errors of the equations were 0.528 year in girls and 0.514 and 0.542 year in boys.

The new equations [13] were based on Canadian youth in the Pediatric Bone Mineral Accrual Study (PBMAS, 1991–7, 79 boys, 72 girls), and cross-validated on Canadian youth from the Healthy Bones Study III (1999–2012, 42 boys, 39 girls) and English youth in the Harpenden Growth Study (1948–71, 38 boys, 32 girls). All youth were of European ancestry. Ages at PHV were estimated with cubic splines: PBMAS, boys 13.4 ± 0.7 years (11.1–15.6 years), girls 11.9 ± 0.7 years (10.3–13.6 years); Healthy Bones Study III, boys 13.5 ± 1.1 years (10.9–15.9 years), girls 11.6 ± 0.7 years (10.5–13.4 years); and Harpenden Growth Study, boys 14.0 ± 1.0 years (11.3–16.2 years), girls 12.1 ± 1.0 years (9.8–14.2 years).

Predictions with the original sex-specific equations (labelled Mirwald) [3] were included for comparison:

$$\begin{aligned} & {\text{Girls:}}\quad {\text{Maturity}}\;{\text{offset}}\;({\text{years}}) = - 9. 3 7 6 + (0.000 1 8 8 2\times ({\text{leg}}\;{\text{length}} \times {\text{sitting}}\;{\text{height}})) \\ & + (0.00 2 2\times ({\text{age}} \times {\text{leg}}\;{\text{length}})) + (0.00 5 8 4 1\times ( {\text{age}} \times {\text{sitting}}\;{\text{height)}}) \\ & - (0.00 2 6 5 8\times ( {\text{age}} \times {\text{mass)}}) + (0.0 7 6 9 3\times ( {\text{mass}}\;{\text{by}}\;{\text{stature}}\;{\text{ratio}} \times 1 00 )); \\ \end{aligned}$$

and

$$\begin{aligned} & {\text{Boys:}}\quad {\text{Maturity}}\;{\text{offset}}\;({\text{years}}) = - 9. 2 3 6 + ((0.000 2 70 8\times ({\text{leg}}\;{\text{length}} \times {\text{sitting}}\;{\text{height}})) \\ & + ( - 0.00 1 6 6 3\times ( {\text{age}} \times {\text{leg}}\;{\text{length)}}) + (0.00 7 2 1 6\times ( {\text{age}} \times {\text{sitting}}\;{\text{height)}}) \\ & + (0.0 2 2 9 2\times ( {\text{mass}}\;{\text{by}}\;{\text{stature}}\;{\text{ratio}} \times 100 )). \\ \end{aligned}$$

The equations were based on three studies: PBMAS, the Saskatchewan Growth and Development Study (1964–73, 71 boys, 40 girls) and the Leuven Longitudinal Twin Study (1985–99, 50 boys, 48 girls). All youth were of European ancestry. Standard errors of the equations were 0.569 year in girls and 0.592 year in boys [3].

Predicted age at PHV (years) was the difference between CA and maturity offset with each equation. Although predicted maturity offset and age at PHV are reported, the analyses focused on predicted age at PHV and the difference of predicted minus observed ages at PHV.

### Analyses

Descriptive statistics (means, standard deviations) were calculated by CA group for actual and predicted maturity offset, predicted age at PHV and the difference of predicted minus observed age at PHV with each equation. The whole year was the midpoint of the range defining CA groups, i.e. 8.0 = 7.50–8.49 years. Corresponding statistics (except for actual offset) were calculated relative to years before/after observed PHV: −3 = −2.51 to −3.50, −2 = −1.51 to −2.50, −1 = −0.51 to −1.50, 0 = −0.50 to +0.49, +1 = +0.50 to +1.49, +2 = +1.50 to +2.49 and +3 = +2.50 to +3.49.

Each individual was also classified as early, average or late maturing using sex-specific mean observed ages at PHV ±1.0 year for the total samples of boys and girls, respectively. The band of 1 year approximated standard deviations for ages at PHV in longitudinal studies [18] and for SA by CA group from 10 to 17 years [19]. Boys and girls with an observed age at PHV within ±1.0 year of the sex-specific mean were thus classified average maturing or on time, i.e. ages at PHV between 13.1 and 15.1 years in boys and between 10.9 and 12.9 years in girls. Boys and girls with an observed age at PHV <13.1 and <10.9 years, respectively, were classified as early maturing, while boys and girls with an observed age at PHV >15.1 and > 12.9 years, respectively, were classified as late maturing. Sample sizes and means and standard deviations for age at PHV in each maturity group were as follows: boys—early, *n* = 36, 12.57 ± 0.41 years; average, *n* = 117, 13.97 ± 0.52 years; late, *n* = 40, 15.69 ± 0.56 years; and girls—early, *n* = 28, 10.19 ± 0.48 years; average, *n* = 140, 11.91 ± 0.52 years; late, *n* = 30, 13.38 ± 0.41 years. Descriptive statistics for each group were calculated by CA and relative to PHV.

Sex-specific analyses were done by CA group and relative to PHV for each equation in the total sample and by maturity groups. Differences of predicted minus observed ages at PHV were tested separately for each equation with two-way analyses of variance; CA groups or years before and after PHV and method (predicted vs. observed) were independent variables. Pairwise comparisons were evaluated with Tukey’s honest significant difference test.

Sex-specific Bland–Altman [20] plots and regressions of the difference of predicted minus observed ages at PHV (*y*-axis) and the mean of predicted and observed ages at PHV (*x*-axis) were performed for each equation by CA group and year before/after PHV. The slopes provided an estimate of bias and limit of agreement between predicted and observed ages at PHV.

Percentages of predicted ages at PHV within ±0.5 year of observed age at PHV for boys and girls in each maturity group were also calculated by CA group and year before/after PHV. The ±0.5 year band approximated the standard errors of the prediction equations. All calculations were done with Statistica 12.0 (Statistica, Tulsa, Oklahoma, USA) [21]. Predicted ages at PHV at each observation were also plotted relative to observed age at PHV for each individual to highlight intra-individual variation.

## Results

### Total Samples

Descriptive statistics and significance of differences of predicted minus observed ages at PHV by CA group are summarised in Tables 1 and 2. Several youth had two observations that fell within an age class; one observation was removed. Several also missed an examination in late adolescence. Predicted maturity offset with each equation is, on average, negative and greatest at 8 years of age and decreases linearly with CA; mean predicted offset approximates zero at 12 years of age in girls and 14 years of age in boys.

**Table 1:** Descriptive statistics for chronological age, age at prediction, actual and predicted maturity offset, predicted ages at peak height velocity (PHV) and differences of predicted age at PHV minus observed age at PHV (criterion) with each equation in boys

***N***	Age, years		Maturity offset, years								Predicted ages at PHV, years						Predicted age at PHV minus observed age at PHV, years					
			Actual^a^		Predicted																	
					Moore-1		Moore-2		Mirwald		Moore-1		Moore-2		Mirwald		Moore-1		Moore-2		Mirwald	
	*M*	SD	*M*	SD	*M*	SD	*M*	SD	*M*	SD	*M*	SD	*M*	SD	*M*	SD	*M*	SD	*M*	SD	*M*	SD
186	8.1	0.3	−5.97	1.14	−4.23	0.23	−4.32	0.22	−4.50	0.29	12.35	0.18	12.43	0.18	12.61	0.26	−1.74	1.09***	−1.65	1.09***	−1.47	1.09***
186	9.0	0.3	−5.04	1.17	−3.68	0.28	−3.75	0.28	−3.95	0.35	12.70	0.20	12.77	0.20	12.97	0.28	−1.36	1.10***	−1.30	1.09***	−1.09	1.09***
185	10.1	0.3	−4.01	1.17	−3.01	0.30	−3.05	0.29	−3.29	0.37	13.06	0.21	13.11	0.22	13.34	0.30	−1.01	1.09***	−0.96	1.09***	−0.73	1.08***
186	11.0	0.3	−3.00	1.13	−2.35	0.30	−2.40	0.31	−2.65	0.38	13.40	0.24	13.44	0.24	13.69	0.34	−0.64	1.05***	−0.59	1.04***	−0.35	1.03**
186	12.0	0.3	−2.07	1.17	−1.74	0.35	−1.71	0.38	−2.07	0.45	13.73	0.27	13.70	0.28	14.06	0.38	−0.33	1.04**	−0.36	1.02***	−0.01	1.02
183	13.0	0.3	−1.07	1.16	−0.94	0.46	−0.86	0.45	−1.25	0.58	13.92	0.35	13.84	0.35	14.23	0.49	−0.13	0.93	−0.21	0.94	0.18	0.87
191	14.0	0.3	−0.07	1.16	−0.03	0.55	0.04	0.52	−0.32	0.70	14.02	0.46	13.95	0.43	14.31	0.49	−0.04	0.84	−0.11	0.89	0.25	0.78*
184	15.0	0.3	0.95	1.14	0.92	0.60	0.96	0.54	0.66	0.76	14.06	0.51	14.03	0.44	14.33	0.68	0.03	0.80	−0.00	0.90	0.30	0.75**
185	16.0	0.3	1.91	1.17	1.80	0.56	1.78	0.49	1.53	0.70	14.17	0.48	14.19	0.41	14.45	0.63	0.11	0.92	0.13	1.03	0.38	0.89***
179	17.0	0.3	2.90	1.17	2.65	0.52	2.55	0.47	2.33	0.63	14.33	0.44	14.43	0.39	14.65	0.57	0.25	1.01	0.35	1.11**	0.57	1.01***
174	18.0	0.3	3.85	1.19	3.41	0.51	3.27	0.48	3.01	0.60	14.56	0.43	14.71	0.41	14.96	0.54	0.43	1.09***	0.58	1.19***	0.82	1.09***

Moore-1: recommended equation, age and sitting height, Moore-2: alternative equation, age and height [13], Mirwald: original equation [3]

*M* mean, *SD* standard deviation

* *p* < 0.05; ** *p* < 0.01; *** *p* < 0.001

^a^Actual maturity offset = observed age at PHV – chronological age at prediction; predicted offset − actual offset = predicted age − observed age at PHV

**Table 2:** Descriptive statistics for chronological age, actual maturity offset, predicted maturity offset and ages at peak height velocity (PHV), and the differences of predicted age at PHV minus observed age at PHV (criterion) with each equation in girls

*N*	Age, years		Maturity offset, years						Predicted age at PHV, years				Predicted age at PHV minus observed age at PHV, years			
			Actual^a^		Predicted											
					Moore-1		Mirwald		Moore-1		Mirwald		Moore-1		Mirwald	
	*M*	SD	*M*	SD	*M*	SD	*M*	SD	*M*	SD	*M*	SD	*M*	SD	*M*	SD
196	8.0	0.3	−3.85	1.04	−3.52	0.26	−3.56	0.35	11.57	0.19	11.60	0.29	−0.33**	0.94	−0.29*	0.91
175	9.0	0.3	−2.90	1.05	−2.84	0.31	−2.88	0.39	11.82	0.22	11.85	0.32	−0.06	0.93	−0.03	0.90
188	10.0	0.3	−1.88	1.05	−2.07	0.36	−2.13	0.45	12.07	0.27	12.13	0.38	0.19	0.89	0.24	0.84
185	11.0	0.3	−0.87	1.03	−1.22	0.39	−1.28	0.48	12.24	0.32	12.29	0.43	0.35***	0.85	0.41***	0.80
181	12.0	0.3	0.25	1.00	−0.28	0.45	−0.36	0.54	12.31	0.37	12.40	0.48	0.43***	0.77	0.50***	0.71
190	13.0	0.3	1.09	1.03	0.63	0.47	0.50	0.53	12.38	0.38	12.51	0.46	0.46***	0.86	0.59***	0.79
196	14.0	0.3	2.12	1.02	1.51	0.45	1.32	0.47	12.50	0.37	12.68	0.41	0.62***	0.95	0.79***	0.88
186	15.0	0.3	3.12	1.05	2.31	0.45	2.04	0.44	12.71	0.38	12.97	0.40	0.81***	1.03	1.08***	0.98
173	16.0	0.3	4.12	1.03	3.03	0.44	2.64	0.41	12.98	0.41	13.35	0.40	1.08***	1.05	1.46***	1.01

Moore-1: recommended equation with age and height [7], Mirwald: original equation [3]

*M* mean, *SD* standard deviation

* *p* < 0.05; ** *p* < 0.01; *** *p* < 0.001

^a^Actual maturity offset = observed age at PHV − chronological age at prediction; predicted offset − actual offset = predicted age − observed age at PHV

Mean predicted ages at PHV increase with CA, but standard deviations are reduced, more so for the new equations. Ranges of predicted ages are also reduced: boys — Moore-1: 0.96–2.52 years, Moore-2: 0.97–2.25, Mirwald: 1.43–3.30 years; and girls — Moore-1: 0.98–2.04 years, Mirwald: 1.57–2.49 years. As noted in Sect. 2, standard deviations and especially ranges for observed ages at PHV are larger.

Relative to PHV, mean predicted ages at PHV with the Moore equations in boys increase from −3 years to PHV and are then relatively stable, while predictions with Mirwald increase from −3 to −1 year of PHV and are then relatively stable (Table 3). Mean differences are negative in boys at −3 and −2 years for the new equations (predicted earlier than observed) but approximate zero at −1 year through +2 years of PHV. The mean difference with Mirwald is negative at −3 years, approaches zero at −2 years, and is then positive and stable from −1 year through +2 years of PHV. Among girls, in contrast, predicted ages at PHV with the new and original equations are similar to observed age at PHV at −3 years and then increase (Table 4). Standard deviations for predicted ages at PHV with the new equations are consistently less than with the original equations in both sexes.

**Table 3:** Descriptive statistics for predicted maturity offset and ages at peak height velocity (PHV), and differences of predicted age at PHV minus observed age at PHV (criterion) with each equation 3 years before to 3 years after observed age at PHV in boys

Years relative to PHV	*N*	Maturity offset, years						Predicted age at PHV, years						Predicted age at PHV minus observed age at PHV, years					
		Moore-1		Moore-2		Mirwald		Moore-1		Moore-2		Mirwald		Moore-1		Moore-2		Mirwald	
		*M*	SD	*M*	SD	*M*	SD	*M*	SD	*M*	SD	*M*	SD	*M*	SD	*M*	SD	*M*	SD
−3	185	−2.35	0.73	−2.37	0.77	−2.67	0.70	13.43	0.53	13.45	0.49	13.74	0.64	−0.63	0.69**	−0.61	0.73***	−0.32	0.66
−2	180	−1.71	0.73	−1.67	0.79	−2.05	0.70	13.77	0.51	13.73	0.46	14.11	0.63	−0.27	0.69	−0.30	0.75*	0.07	0.67
−1	184	−0.96	0.77	−0.87	0.84	−1.31	0.73	14.02	0.51	13.93	0.47	14.37	0.64	−0.02	0.73	−0.11	0.78	0.32	0.68*
0	179	−0.01	0.82	0.09	0.87	−0.32	0.80	14.07	0.52	13.96	0.49	14.38	0.67	0.02	0.76	−0.08	0.81	0.33	0.73*
1	188	1.03	0.85	1.09	0.89	0.78	0.83	14.02	0.54	13.96	0.50	14.27	0.70	−0.04	0.79	−0.10	0.82	0.21	0.75
2	187	1.94	0.85	1.91	0.88	1.69	0.81	14.08	0.55	14.11	0.51	14.33	0.72	0.06	0.80	0.09	0.84	0.31	0.76
3	177	2.73	0.82	2.62	0.87	2.43	0.78	14.26	0.55	14.37	0.51	14.56	0.71	0.26	0.79	0.37	0.83**	0.56	0.75

Moore-1: recommended equation, age and sitting height, Moore-2: alternative equation, age and height [13], Mirwald: original equation [3]

*M* mean, *SD* standard deviation

* *p* < 0.05; ** *p* < 0.01; *** *p* < 0.001

**Table 4:** Descriptive statistics for predicted maturity offset and ages at peak height velocity (PHV), and differences of predicted age at PHV minus observed age at PHV (criterion) with each equations 3 years before to 3 years after observed age at PHV in girls

Years relative to PHV	*N*	Maturity offset, years				Predicted age at PHV, years				Predicted age at PHV minus observed age at PHV, years			
		Moore-1		Mirwald		Moore-1		Mirwald		Moore-1		Mirwald	
		*M*	SD	*M*	SD	*M*	SD	*M*	SD	*M*	SD	*M*	SD
−3	176	−2.78	0.58	−2.84	0.57	11.89	0.38	11.95	0.47	−0.11	0.56	−0.17	0.57
−2	181	−2.13	0.70	−2.20	0.67	12.07	0.41	12.14	0.52	0.17	0.65	0.23	0.61
−1	187	−1.32	0.77	−1.39	0.71	12.25	0.42	12.33	0.55	0.37*	0.72	0.44*	0.65
0	188	−0.37	0.80	−0.46	0.72	12.31	0.42	12.41	0.56	0.41*	0.74	0.50*	0.65
1	187	0.61	0.84	0.48	0.73	12.33	0.42	12.45	0.54	0.42*	0.78	0.54*	0.67
2	196	1.49	0.81	1.33	0.69	12.45	0.45	12.62	0.57	0.54*	0.77	0.71*	0.65
3	181	2.22	0.81	1.96	0.67	12.65	0.46	12.88	0.56	0.82*	0.78	1.07*	0.65

Moore-1: recommended equation, age and height [13], Mirwald: original equation [3]

*M* mean, *SD* standard deviation

* *p* < 0.001

Slopes of the sex-specific Bland–Altman regressions for each prediction equation are negative and significant, and indicate consistent bias by CA group [Table 1 of the Electronic Supplementary Material (ESM)] and by years relative to PHV (Table 2 of the ESM). The slopes are greater for the new equations at all ages in boys and from 8 to 14 years in girls, and from −3 through +3 years of PHV in both sexes.

### Contrasting Maturity Groups

**Fig. 1:**
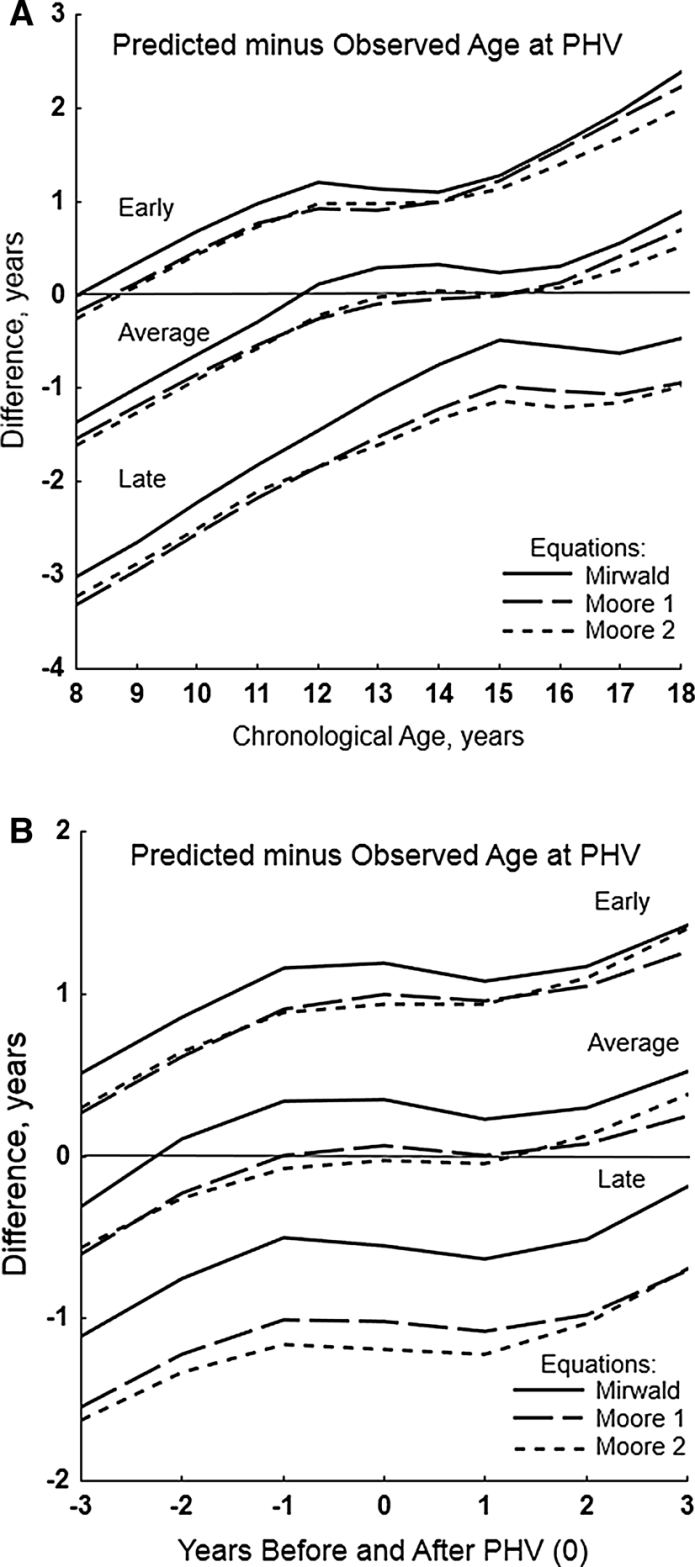
Means of predicted age at peak height velocity (PHV) minus observed age at PHV with the new (Moore-1, Moore-2) and original (Mirwald) equations in early, average and late maturing boys by age group (**a**) and years before and after PHV (**b**); standard errors of the means ranged from 0.03 to 0.12 year

**Fig. 2:**
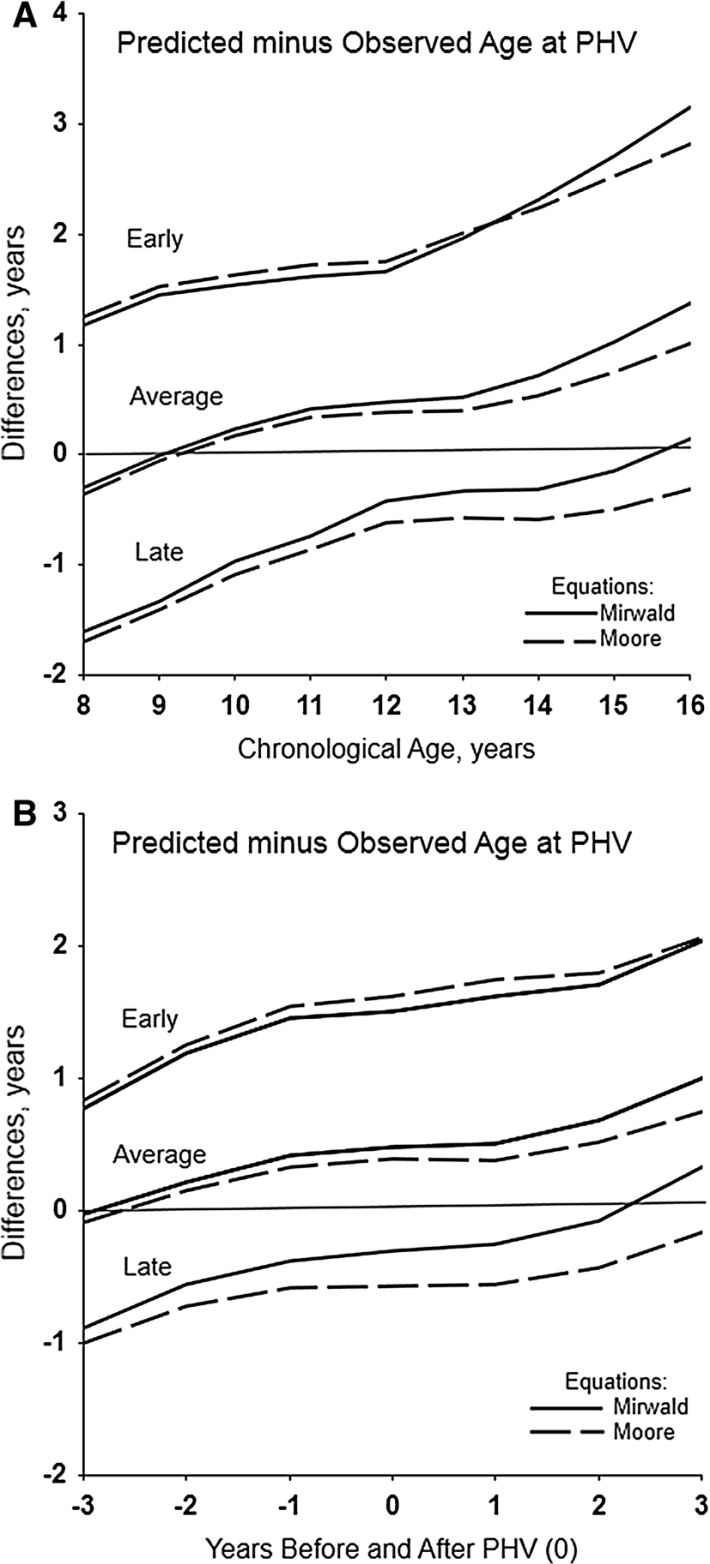
Means of predicted age at peak height velocity (PHV) minus observed age at PHV with the new (Moore-1) and original (Mirwald) equations in early, average and late maturing girls by age group (**a**) and years before and after PHV (**b**); standard errors of the means ranged from 0.04 to 0.12 year

Mean differences of predicted minus observed ages at PHV for each equation in youth of contrasting maturity status are shown by CA and relative to PHV in Fig. 1 (boys) and Fig. 2 (girls). Corresponding descriptive statistics and significance of differences are summarised in Tables 3 and 4 of the ESM.

Mean differences of predicted minus observed ages at PHV with the new and original equations are positive and increase with CA in early maturing boys (except at 8 years) and girls, and are negative and increase with CA in late maturing boys and girls (except at 16 years). Predicted ages at PHV are later than observed age at PHV in early maturing and earlier than observed age at PHV in late maturing boys and girls. The mean differences are similar between the Moore equations and vary only slightly from Mirwald in early and late maturing boys (Fig. 1a). Corresponding differences between equations are similar in early maturing girls, but are larger for Moore than Mirwald in late maturing girls (Fig. 2a).

Among average maturing boys, mean differences of predicted minus observed ages at PHV are negligible between Moore-1 and Moore-2. Predicted ages are less than observed age at PHV from 8 to 12 years, about equivalent at 13–15 years, and greater from 16 to 18 years. For Mirwald, predicted ages are less than observed age at PHV from 8 to 11 years, about equal at 12 years and greater from 13 to 18 years (Fig. 1a). Among average maturing girls, predicted ages at PHV are slightly earlier at 8 years, approximate observed age at PHV at 9 years and are later at 10–16 years (Fig. 2a). Differences of predicted minus observed ages at PHV between Moore-1 and Mirwald are small from 8 to 12 years but increase with age.

Relative to years before/after PHV, mean differences of predicted minus observed ages at PHV are similar for the Moore equations and less than those for Mirwald in early maturing boys; the differences are positive (predicted later than observed) and increase from −3 to +3 years of PHV (Fig. 1b). The corresponding differences are similar for Moore-1 and Mirwald among early maturing girls, and are positive and increase from −3 to +3 years (Fig. 2b).

Differences of predicted minus observed ages at PHV are negative (predicted earlier than observed) from −3 to +3 years of PHV in late maturing boys; predicted ages at PHV with the Moore equations are markedly earlier than observed age at PHV. Corresponding differences among late maturing girls are also negative; predicted ages at PHV are earlier than observed ages, more so with Moore-1 than Mirwald, except at +3 years.

Among average maturing boys, differences of predicted minus observed ages at PHV are negligible between Moore equations; the differences are negative at −3 and −2 years, approximate zero from −1 year to +2 years and increase at +3 years (Fig. 1b). Corresponding differences with Mirwald are negative at −3 years, near zero at −2 years and then positive. In contrast, mean differences of predicted minus observed ages at PHV in average maturing girls approximate zero at −3 years with both equations (Fig. 2b), and are then positive and increase through +3 years (predicted later than observed).

Percentages of predicted ages at PHV within ±0.5 year of observed age at PHV in youth of contrasting maturity status are summarised by CA and relative to PHV in Tables 5 and 6, respectively. Predictions within ±0.5 year of observed age at PHV are highest at 9 years of age and at −3 years of PHV in early maturing boys and then decline by CA and relative to PHV; few predictions are within ±0.5 year of observed PHV from −1 through +3 years of PHV. By CA and relative to PHV, only one prediction (with Mirwald) is within ± 0.5 year of observed PHV in early maturing girls. The trends are generally similar for late maturing boys, especially with the new equations, while percentages of predictions within ±0.5 year of observed age at PHV overlap for the new and original equations among late maturing girls.

**Table 5:** Percentages of predicted ages at peak height velocity (PHV) within ±0.5 year of observed age at PHV with the new: Moore-1 (M-1), Moore-2 (M-2) and original: Mirwald (M) equations by age at prediction in boys and girls of contrasting maturity status

Age group	Boys									Girls					
	Early (*n* = 32–36)			Average (*n* = 106–115)			Late (*n* = 37–40)			Early (*n* = 23–28)		Average (*n* = 123–140)		Late (*n* = 25–30)	
	M-1	M-2	M	M-1	M-2	M	M-1	M-2	M	M-1	M	M-1	M	M-1	M
8	66	75	72	0	1	3	0	0	0	0	4	54	56	0	0
9	79	79	71	7	12	19	0	0	0	0	0	58	64	0	0
10	66	66	37	28	31	43	0	0	0	0	0	66	65	3	10
11	31	25	11	46	50	60	0	0	0	0	0	58	55	14	32
12	3	11	3	66	63	63	0	0	0	0	0	59	51	46	65
13	0	9	0	71	71	63	0	0	16	0	0	57	47	53	77
14	0	8	3	78	74	62	5	3	40	0	0	46	34	52	76
15	0	0	0	73	68	62	11	11	51	0	0	37	20	67	79
16	0	0	0	63	62	60	13	8	55	0	0	24	6	65	60
17	0	0	0	56	45	45	14	14	49						
18	0	0	0	45	34	30	23	26	56						

M-1: boys—recommended equation, age and sitting height, girls—recommended equation, age and height; M-2: boys—alternative equation, age and height [13]; M: original equation [3]

**Table 6:** Percentages of predicted ages at peak height velocity (PHV) within ±0.50 year of observed age at PHV with the new: Moore-1 (M-1), Moore-2 (M-2) and original: Mirwald (M) equations by years before and after PHV in boys and girls of contrasting maturity status

Years relative to PHV	Boys									Girls					
	Early (*n* = 34–37)			Average (*n* = 109–116)			Late (*n* = 33–38)			Early (*n* = 13–27)		Average (*n* = 129–140)		Late (*n* = 20–30)	
	M-1	M-2	M	M-1	M-2	M	M-1	M-2	M	M-1	M	M-1	M	M-1	M
−3	80	80	51	43	50	71	0	0	11	0	0	81	81	3	14
−2	43	40	14	76	71	75	3	0	24	0	4	72	71	22	41
−1	3	8	3	79	77	66	11	11	50	0	0	62	58	52	69
PHV	0	6	0	76	75	58	11	8	58	0	0	59	49	55	76
+1	0	9	3	72	73	66	13	8	45	0	0	57	47	53	79
+2	0	3	0	72	66	59	19	22	54	0	0	49	36	67	83
+3	0	0	0	60	49	47	36	36	58	0	0	38	13	77	45

M-1: boys—recommended equation, age and sitting height, girls—recommended equation, age and height; M-2: boys—alternative equation, age and height [13]; M: original equation [3]

Among average maturing boys, percentages of predicted ages at PHV within ±0.5 year of observed age at PHV with Moore-1 and Moore-2 increase from 8 to 14 years and then decline, while percentages with Mirwald are rather stable (~60%) from 11 to 16 years (Table 5). Among average maturing girls, corresponding percentages with Moore-1 and Mirwald are highest at 10 years (~65%) and then decline. Predictions within ± 0.5 year of observed age at PHV are rather stable from −2 to +2 years of PHV with the new equations (~71–79%) in average maturing boys; percentages with Mirwald are highest at −2 years but then decline (Table 6). In contrast, percentages are highest at −3 years of PHV with both equations (81%) and then systematically decline in average maturing girls.

### Intra-Individual Variation

**Fig. 3:**
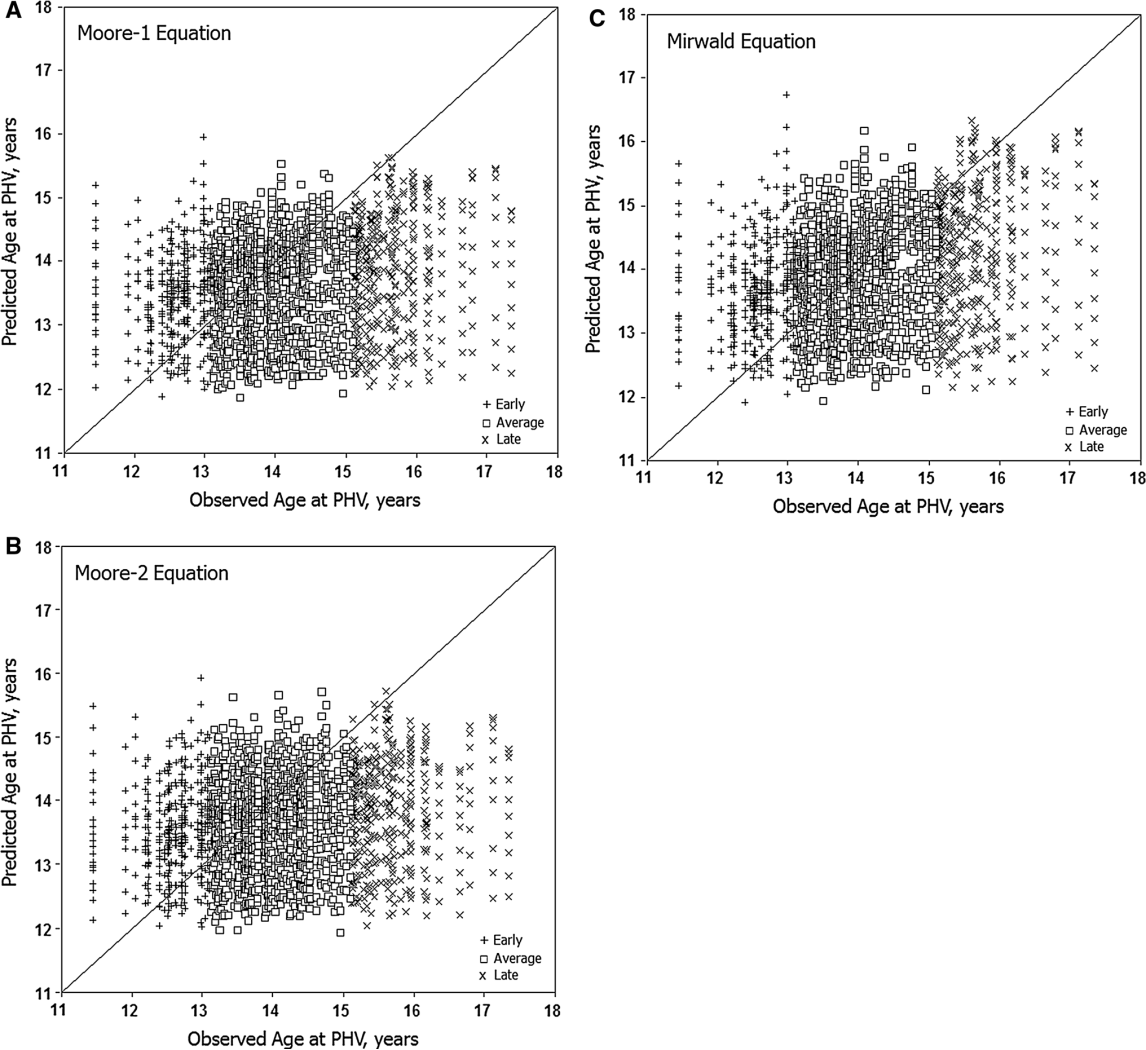
Predicted ages at peak height velocity (PHV) in boys with the Moore-1 (**a**), Moore-2 (**b**) and Mirwald (**c**) equations plotted relative to observed age at PHV at each age for individual early, average and late maturing boys. Each *vertical array* shows predicted ages at PHV for an individual. The *diagonal line* indicates that predicted age at PHV is equivalent with observed age at PHV

**Fig. 4:**
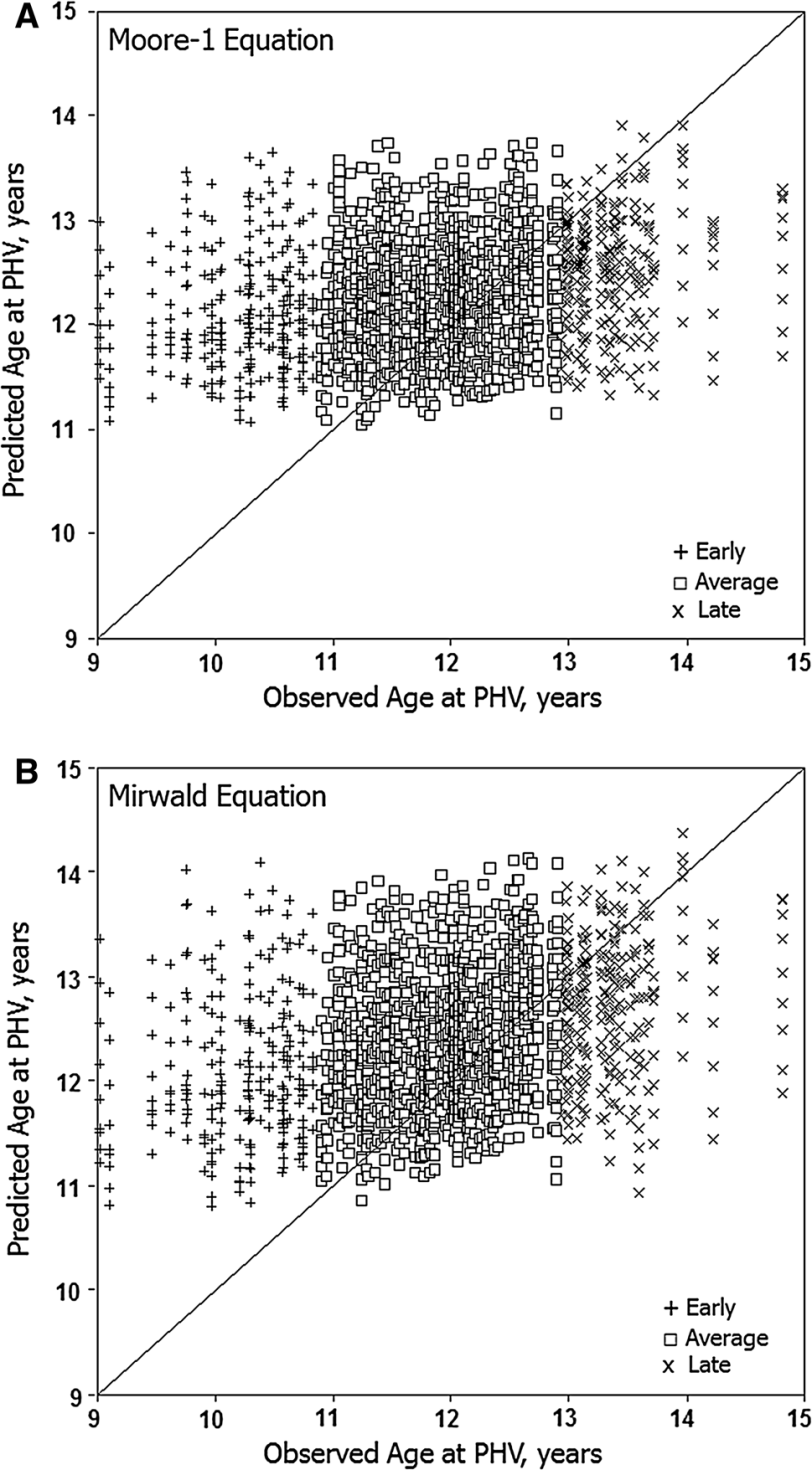
Predicted ages at peak height velocity (PHV) with the Moore-1 (**a**) and Mirwald (**b**) equations plotted relative to observed age at PHV at each age for individual early, average and late maturing girls. Each *vertical array* shows predicted ages at PHV for an individual. The *diagonal line* indicates that predicted age at PHV is equivalent with observed age at PHV

Predicted ages at PHV (*y*-axis) for individual youth with each equation are illustrated relative to observed ages at PHV (*x*-axis) in Fig. 3 (boys) and Fig. 4 (girls). Intra-individual variation in predicted ages at PHV is considerable and ranges of predicted ages are reduced with the new equations. Relatively few predicted ages approximate observed ages at PHV in early and late maturing boys and girls.

## Discussion

Results of the validation analysis of the new maturity offset prediction equations [13] were consistent with those for the original equations in Polish [10, 11] and American [12] youth. The combined results of the four analyses indicated several trends:

First, predicted maturity offset decreased and age at PHV increased, on average, with CA at prediction throughout the range considered. This likely reflected the dependence of the predictions upon CA and probably body size at prediction.

Second, variation in predicted ages at PHV within CA groups was reduced compared with variation in observed ages at PHV. Moreover, variation with the new equations was reduced compared with that for the original equations.

Third, predictions of age at PHV were influenced by observed or actual age at PHV, especially among early and late maturing youth. Predicted ages at PHV with the new and original equations were consistently later than observed age at PHV among early maturing, and earlier than observed age at PHV in late maturing youth of both sexes.

Fourth, predicted ages at PHV with the new equations were quite accurate for average maturing boys *within ±1* *year of observed PHV*. A corresponding window for average maturing girls was not evident.

Fifth, intra-individual variation in predicted ages at PHV with the modified and original equations was considerable. Relatively few predicted ages at PHV in early and late maturing boys and girls approximated observed age at PHV.

Sixth, slopes of the Bland–Altman regressions for all predictions were negative by CA and year from −3 to +3 years of PHV in both sexes, and indicated a bias between predicted and observed ages at PHV. Slopes were greater for the new compared with the original equations.

Prior to discussing implications of the present validation study, the labels maturation, maturity status and maturity timing require clarification as they are often treated as synonymous. Maturation is a process, specifically, of progress towards the biologically mature state, which varies among bodily systems. Maturity status refers to the level or state of maturation at the time of observation. Maturity timing refers to the age at which specific maturational events occur [2]. Age at PHV is an indicator of maturity timing—the estimated age at maximal velocity of growth in height during the adolescent spurt. Age at PHV is often discussed relative to SA and stage of puberty (secondary sex characteristics), which are indicators of maturity status. Though related, maturity timing and status are not equivalent, and variation in maturity status at the time of PHV is considerable. In the Wroclaw Growth Study, for example, SA at the time of PHV ranged from 9.6 to 14.2 years in girls and from ~12.5 to 15.5 years in boys [22, 23]. In the Zurich longitudinal study, four of the five stages of pubic hair and all five stages of breast development in girls, and all stages of pubic hair and four of the five stages of genital development in boys were evident at the time of PHV [24, 25].

Results of the validation analyses have implications for those working with youth athletes. Identification of the interval of PHV is central in the late specialisation component of the long-term athlete development model [4, 5], while prediction of maturity offset has been discussed in the context of individualising training relative to the timing of the growth spurt [6–8]. However, potential misclassifications and implications of misclassification for individual youth are not considered.

The potential utility of predicted maturity offset as a categorical indicator of maturity status, specifically pre- and post-PHV, was emphasised in the initial descriptions of the equations [3, 13]. Given results of the present (Figs. 1, 2; Tables 5, 6) and earlier validation analyses [10–12], this is likely useful with average maturing boys in single-year CA groups close to the time of PHV, age ~13–15 years. Unfortunately, the maturity status of youth is not ordinarily known when the equations are applied. A corresponding window for average maturing girls is not apparent, while predictions in early and late maturing youth of both sexes have limited utility for classification purposes.

Classifications of youth as pre- and post-PHV may have merit within a narrow CA range, but CA and body size are potential confounders. Among soccer players aged 9.2–10.4 years classified into CA quartiles, maturity offset decreased while height and predicted age at PHV increased from the youngest to oldest quartiles [26]. The influences of CA and size on maturity classifications are more marked in youth spanning several years across adolescence. Among male soccer players aged 11–17 years [27] and 10–18 years [28], sport academy participants aged 11–15 years [29] and school boys aged 11–15 years [30], CA, height and weight increased, on average, from pre- to mid- to post-PHV groups, or by years before or after predicted PHV. The approach was similar to grouping boys by stage of pubic hair or genital development independent of CA [31]. It is likely that 11- and 14-year-old boys classified as pre-PHV or 11- and 15-year-old boys classified as mid-PHV differed in height, weight and perhaps performance.

As noted, grouping youth by maturity status is central to many talent development programmes, but reduced variation in predicted ages at PHV (Tables 1, 2) limits its utility. For example, youth soccer players aged 11–15 years were classified as early, average and late maturing on the basis of SA and predicted age at PHV. Maturity status was classified by the differences of SA minus CA, and of predicted age at PHV and the average age at PHV of the samples used to develop the original equation [32]. Concordance of maturity classifications (kappa coefficients) based on SA and predicted age of PHV was relatively poor in two age groups of players. Among 87 players aged 11.0–12.9 years, 84 (97%) were classified as average by predicted age at PHV compared with 45 (52%) by SA. Among 93 players aged 13.3–15.3 years, 77 (83%) were classified as average by predicted age at PHV compared with 55 (59%) by SA [32]. The high percentages of average maturing players reflected the reduced range of variation in predicted ages at PHV.

Predicted offset and/or age at PHV does not effectively discriminate between early and late maturing boys, i.e. it over- and under-estimates age at PHV in early and late maturing boys, respectively (Fig. 1), with the result that players are essentially regressed to the mean age of PHV. The preceding highlights limitations of using predicted ages at PHV (maturity timing) to classify youth athletes into maturity status categories, currently labelled bio-banding [9]. The offset method has been used to bio-band soccer players for competition [33], but noting the limitations of predicted offset, bio-banded tournaments organised by the English Premier League have used, with some success, an alternative non-invasive predictor of maturity status—percentage of predicted adult height attained at the time of observation [9]. Specifics of the method [2] and its application are beyond the scope of this discussion [9].

Variation in the skeletal maturity status of youth male athletes in several sports has relevance for the preceding discussion. Allowing for variation among methods of SA assessment, reasonably similar proportions of late, average and early maturing boys were represented in sport-specific samples of athletes at 11–12 years of age. With increasing CA, proportions of late maturing players declined while proportions of average and early maturing players increased. Moreover, several 14-year-old male athletes were skeletally mature, and the number of mature athletes increased with CA [19]. These trends have implications for classifications based on predicted offset or age at PHV, especially at the maturity extremes.

Corresponding observations for female athletes are limited. Predicted age at PHV was used as an indicator of maturity status in a study of performance and motor coordination of gymnasts 6–8 years of age [34]. Mean predicted ages at PHV increased from 6 (11.2 ± 0.2) to 8 (11.6 ± 0.2) years, but were considerably earlier and had reduced standard deviations compared with observed ages at PHV in Belgian (12.9 ± 1.5 years) and Polish (13.2 ± 0.9 years) gymnasts [35] and predicted age at PHV in a mixed-longitudinal sample of English gymnasts, 13.1 ± 0.7 years [36]. The latter estimate was likely influenced by CA range of the sample (8–17 years of age). Limitations of the original prediction equations with the sample of Belgian gymnasts have been previously reported [37]. The relatively early predicted ages at PHV among gymnasts 6–8 years old also contrasted skeletal maturity observations. Skeletal age and CA did not differ, on average, among gymnasts aged 5–10 years; with increasing CA, SA lagged behind CA and the proportion of late maturing girls increased [19].

The adolescent growth spurt is often indicated as a risk factor for injury [38]. For example, the peak prevalence of Sever’s disease (inflammation of the growth plate of the calcaneus) and Osgood–Schlatter’s disease (inflammation of the patellar tendon of the anterior quadriceps muscle at the tibial tuberosity) occurred, respectively, in U-11 and U-13–U-14 academy soccer players, leading the authors to emphasise “… the importance to football clubs of identifying the onset of these growth spurts to start early effective treatment and management and even prevention of these injuries” [39, p. 469–70]. The specific growth spurts were not identified. The two conditions accounted for 5% of all injuries among U-9–U-19 players; most Sever’s (~84%) and Osgood–Schlatter’s (~87%) inflammations occurred among U-10–U-14 players and U-12–U-16 players, respectively. Both conditions are often attributed to rapid growth of the foot, lower leg (tibia) and thigh (femur), which occurs early in the male adolescent spurt [40].

Predicted age at PHV was related to injuries among 26 soccer players (11.9 ± 0.84 years at initial selection) followed for 3 years [41]. The mean number of traumatic and overuse injuries per player were lower among pre-PHV players, but did not differ between players at PHV and post-PHV. A subsequent analysis suggested an increase in overuse injuries among players with an older age at PHV [42].

The growth and maturity status of injured athletes are not ordinarily reported. What specifically about the adolescent spurt renders a youngster at risk for injury? Is it the early acceleration of growth rate at take-off of the spurt or the peak velocity of growth at PHV? Is it the early rapid growth of the lower extremities? The differential timing of growth spurts in body segments (foot and leg length, sitting height), bone area and bone mineral, muscle mass and muscular strength and power [40] needs attention. Other factors including behavioural changes during adolescence and training environments per se [43] merit more detailed consideration in the context of injury risk.

Applications of maturity offset/predicted age at PHV are largely limited to the original equations [3]. Both have been used to classify youth into maturity groups and as a covariate or predictor in studies of athletes and non-athletes spanning late childhood through adolescence [1]. Dependence of offset on CA at prediction is problematic; e.g. correlations between CA and maturity offset were high in girls 11–16 years of age, 0.89 [44] and boys 8–18 years of age, 0.97 [45]. Among male soccer players 11–15 years of age, predicted offset with the new and original equations was highly correlated with CA (0.96, Moore-1; 0.96, Moore-2; 0.92, Mirwald) and with height (0.95, Moore-2; 0.94, Mirwald) and sitting height (0.93, Moore-1) (unpublished, based on data in [32]).

Published applications of the new prediction equations [13] are limited. Maturity offset (using CA and height) was described in small samples of U-13 and U-15 soccer players participating in different training programmes, but was not considered in the analyses [14–16]. Predicted ages at PHV (using CA and height) did not differ between ranked and unranked elite U-14, U-15 and U16 tennis players of both sexes, but were a significant predictor of power in male though not in female athletes [17].

Age at PHV is a heritable characteristic as evident in several longitudinal studies of monozygotic (MZ) and dizygotic (DZ) twins [46–50]. Allowing for variation in methods of estimation, heritabilities for age at PHV were relatively high, 0.64–0.93, and implied a significant role for genotype. Heritabilities are sample statistics and may not apply to individuals. Nevertheless, intra-individual variability in predicted ages at PHV (Figs. 3, 4), and variation in predicted ages at PHV with CA per se (Tables 1, 2) and maturity status (Figs. 1, 2) should be considered in this context.

Genetic tallness or shortness may also bias predictions. Heritabilities for stature are relatively high in samples of well-nourished twins, 0.69–0.96, and are similar for segment lengths [51]. Given two youngsters of the same CA, predicted offset and in turn age at PHV will likely differ between genetically tall and short individuals.

The new equations were simplified by reducing potential collinearity given the highly correlated predictors in the original equations (see Sect. 2). However, the standard errors were not appreciably reduced, 0.569–0.528 year in girls, and 0.592–0.514 (Moore-1) and 0.542 (Moore-2) in boys [3, 13].

The present study was not without limitations. Although conditions in Poland during the Wrocław study (1961–72) were not on par with Western countries, mean statures of boys [52] were comparable with contemporary Belgian and Canadian (Ontario) boys, but slightly shorter than UK boys [53] and Canadian (Saskatchewan) [54] boys. Mean statures of Wrocław girls [55] were shorter than Belgian, Canadian (Ontario, Saskatchewan) and UK girls [53, 54]. Sitting height/stature ratios of Wrocław boys and girls, however, overlapped with those for several European samples [53], while leg length/sitting height ratios overlapped with those of Canadian youth in the PBMAS (Table 5 in the ESM).

Secular variation is a related consideration. Heights of European youth increased over time after World War II, but have since slowed or stopped in many countries [56–60]. Median heights of US youth have not changed appreciably since the 1960s [60, 61]. However, secular increases in height were not necessarily associated with accelerated maturation between 1960 and 1980 in Belgium [56, 62] and between 1980 and 1997 in the Netherlands [63].

Mean ages at PHV of Wrocław boys (14.1 ± 1.1 years) and girls (11.9 ± 1.0 years) based on the Preece–Baines 1 model (this study) were similar to graphic estimates for Wrocław twins followed in 1967–83: boys—MZ 14.0 ± 0.9 years, DZ 14.0 ± 1.0 years; girls—MZ 11.8 ± 1.0 years, DZ 11.8 ± 1.1 years [64]. The Preece–Baines 1 model estimates for the male twins were similar, MZ 14.2 ± 0.7 years and DZ 14.1 ± 0.9 years [48]. Ages at PHV of Warsaw boys and girls followed in 1974–82 were 13.8 ± 1.3 and 11.8 ± 0.7 years, respectively [65], and of Poznań boys and girls followed in 1985–98 were 13.9 ± 0.8 and 11.8 ± 0.9 years, respectively [66]. Ages at menarche were also generally similar in the Polish samples, Wrocław Growth Study, 13.2 ± 1.0 years [11], Wrocław twins, MZ 13.1 ± 1.0 years, DZ 13.1 ± 1.1 years [67], Warsaw, 12.9 ± 0.8 years [65] and Poznań, 12.9 ± 0.9 years [66].

Mean ages at PHV in the Polish studies were similar to means in other European longitudinal studies, including Belgian boys (14.2 ± 0.8 years) and girls (12.4 ± 0.8 years), and UK boys (14.0 ± 1.0 years) and girls (12.1 ± 1.0 years) used, respectively, in developing the original [3] and calibrating the new [13] prediction equations. Mean ages at PHV in European and North American longitudinal studies spanning the 1960s through 1990s varied between 13.3 and 14.4 years in boys and 11.3 and 12.2 years in girls [18, 40], and evidence for secular change over the past two generations was inconsistent [68]. Ages at PHV in Danish youth born in the 1930s through 1960s declined, from 12.5 to 12.0 years in girls and from 14.5 to 14.2 years in boys [69], while ages at PHV among American boys and girls born in the 1960s through 1980s did not differ [12].

Most information on secular change in maturity timing is based on age at menarche. Mean ages declined in European girls after World War II, but the declines were largely associated with reductions in the 90th percentiles rather than medians and 10th percentiles [57]. Evidence for recent secular change in menarche and pubertal onset and progress in USA was inconclusive [70–72].

Samples comprising longitudinal studies may or may not be representative of the population in general. Methods for estimating ages at PHV also vary [73]. No one method is the standard; all have underlying assumptions and limitations. Ages at PHV in Wrocław youth were estimated with the Preece–Baines Model 1; standard errors of the model fit compared favourably with other studies [66, 74]. Ages at PHV in samples used to develop the new prediction equations were estimated with interpolating cubic splines in youth with “sufficient height measurements” (boys: 5 between 11.5 and 16.5 years; girls: 4 between 11.0 and 13.0 years). Running velocities were calculated; an interpolating cubic spline was fit “… in a regular grid to identify maximum height velocity …” (and) based on visual selection, “… those that had clear peaks during the pubertal spurt as well as data pre-and post-APHV …” were selected [13, p. 1757].

## Conclusion

The present study validated the new maturity offset prediction equations [13] in 193 boys and 198 girls from the Wrocław Growth Study. Comparisons with the original equations [3] were also included. The new equations were useful for average maturing boys close to the time of PHV; a window for average maturing girls was not apparent. The new and original equations were not useful for early and late maturing boys and girls. Predicted offset and ages at PHV also had reduced ranges of variation compared with observed ages at PHV, more so with the new than the original equations.

Studies applying predicted offset and/or age at PHV are increasing as researchers attempt to address inter-individual differences in biological maturation. Attention to the details of maturity status and timing and to intra- and inter-individual variability in status and timing is essential. Care in use of the equations and awareness of their limitations are also essential. Further validation and the development, refinement and evaluation of alternative approaches to maturity assessment are needed.

## Electronic supplementary material

Below is the link to the electronic supplementary material.

**Supplementary Table 1A** Intercepts and slopes of Bland–Altman regressions of the difference between predicted minus observed ages at peak height velocity (PHV) [y-axis] on the mean of predicted and observed ages at PHV (x-axis) for the three prediction equations in boys by chronological age group. **Supplementary Table 1B** Intercepts and slopes of Bland–Altman regressions of the difference between predicted minus observed ages at peak height velocity (PHV) [y-axis] on the mean of predicted and observed ages at PHV (x-axis) for the two prediction equations in girls by chronological age group (DOCX 18 kb)

**Supplementary Table 2A** Intercepts and slopes of Bland–Altman regressions of the difference between predicted minus observed ages at peak height velocity (PHV) [y-axis] on the mean of predicted and observed ages at PHV (x-axis) for the three prediction equations in boys by years before and after observed age at PHV. **Supplementary Table 2B** Intercepts and slopes of Bland–Altman regressions of the difference between predicted minus observed ages at peak height velocity (PHV) [y-axis] on the mean of predicted and observed ages at PHV (x-axis) for the two prediction equations in girls three years before to 3 years after observed age at PHV (DOCX 17 kb)

**Supplementary Table 3A** Descriptive statistics for actual maturity offset, predicted maturity offset and ages at peak height velocity (PHV), and the difference of predicted minus observed age at PHV with the three prediction equations in early, average and late maturing boys by age group. **Supplementary Table 3B** Descriptive statistics for actual maturity offset, predicted maturity offset and ages at peak height velocity (PHV), and the difference of predicted minus observed age at PHV with the two equations in early, average and late maturing girls by age group (DOCX 30 kb)

**Supplementary Table 4A** Descriptive statistics for predicted maturity offset and ages at peak height velocity (PHV), and the difference of predicted age at PHV minus observed age at PHV for the three equations in early, average and late maturing boys from −3 to +3 years of observed age at PHV. **Supplementary Table 4B** Descriptive statistics for predicted maturity offset and ages at peak height velocity (PHV), and the difference of predicted age at PHV minus observed age at PHV for the two equations in early, average and late maturing girls from −3 to +3 years of observed age at PHV (DOCX 23 kb)

**Supplementary Table 5** Sample sizes and means and standard deviations for the ratio of leg length to sitting height (%) by years before and after observed peak height velocity (PHV) in boys and girls from the Wroclaw Growth Study (WGS) and from the Pediatric Bone Mineral Accrual Study (PBMAS) (DOCX 14 kb)
