# Supplementary material for: Modified Maturity Offset Prediction Equations: Validation in Independent Longitudinal Samples of Boys and Girls
Source: Sports Med. 2017 Jun 12;48(1):221–36. doi: 10.1007/s40279-017-0750-y (PMC5752743; doi:10.1007/s40279-017-0750-y)
Supplement: Supplementary file 1 — Supplementary Table 1A Intercepts and slopes of Bland–Altman regressions of the difference between predicted minus observed ages at peak height velocity (PHV) [y-axis] on the mean of predicted and observed ages at PHV (x-axis) for the three prediction equations in boys by chronological age group. Supplementary Table 1B Intercepts and slopes of Bland–Altman regressions of the difference between predicted minus observed ages at peak height velocity (PHV) [y-axis] on the mean of predicted and observed ages at PHV (x-axis) for the two prediction equations in girls by chronological age group (DOCX 18 kb) [file 40279_2017_750_MOESM1_ESM.docx]

Supplementary Table 1A. Intercepts and slopes of Bland-Altman regressions of the difference between predicted age at PHV minus observed age at PHV (*y*-axis) and the mean of predicted and observed ages at PHV (*x*-axis) for the three prediction equations* in boys by chronological age group

Age, Moore-1 Moore-2 Mirwald

yrs N Intercept SE Slope SE Intercept SE Slope SE Intercept SE Slope SE

8 186 21.26 0.67 -1.72 0.05^a^ 22.00 0.59 -1.78 0.04 20.67 0.79 -1.66 0.06

9 186 21.20 0.71 -1.67 0.05 22.05 0.61 -1.79 0.04 20.49 0.83 -1.60 0.06

10 184 20.85 0.73 -1.60 0.05 21.90 0.66 -1.68 0.05 19.99 0.85 -1.51 0.06

11 185 19.99 0.81 -1.49 0.06 21.29 0.72 -1.59 0.05 18.90 0.94 -1.39 0.07

12 184 18.89 0.77 -1.37 0.05 19.89 0.71 -1.46 0.05 17.52 0.89 -1.25 0.06

13 183 14.99 0.68 -1.07 0.04 16.66 0.71 -1.21 0.05 13.05 0.78 -0.91 0.05

14 191 11.63 0.64 -0.82 0.05 14.23 0.70 -1.02 0.05 9.22 0.73 -0.63 0.05

15 184 10.32 0.68 -0.73 0.05 14.17 0.82 -1.01 0.05 7.86 0.78 -0.53 0.05

16 185 12.19 0.83 -0.85 0.05 17.40 0.99 -1.22 0.07 10.04 0.94 -0.68 0.07

17 179 14.68 1.09 -1.00 0.08 20.67 1.25 -1.43 0.09 12.97 1.22 -0.86 0.09

18 173 16.98 1.26 -1.14 0.09 22.70 1.46 -1.53 0.10 15.58 1.39 -1.01 0.10

^a^All slopes are significant, p<0.001

*Moore-1: recommended equation, age and sitting height, Moore-2: alternative equation, age and height [13]; Mirwald: original equation [3]

Supplementary Table 1B. Intercepts and slopes of the Bland-Altman regressions of the difference between predicted age at PHV minus observed age at PHV (*y*-axis) on the mean of predicted and observed ages at PHV (*x*-axis) for the two prediction equations* in girls by chronological age group

Age, Moore-1 Mirwald

yrs N Intercept SE Slope SE Intercept SE Slope SE

8 196 18.80 0.49 -0.94 0.02^a^ 15.74 0.64 -0.87 0.03

9 175 18.30 0.59 -0.92 0.03 15.20 0.74 -0.84 0.04

10 188 16.62 0.58 -0.90 0.03 13.32 0.68 -0.82 0.04

11 185 15.01 0.59 -0.88 0.04 11.90 0.66 -0.79 0.05

12 181 12.95 0.63 -0.83 0.04 9.85 0.65 -0.74 0.05

13 190 13.94 0.73 -0.80 0.04 11.26 0.70 -0.74 0.05

14 196 15.96 0.90 -0.78 0.05 13.81 0.84 -0.74 0.05

15 186 17.66 1.08 -0.75 0.05 16.33 1.01 -0.75 0.05

16 173 18.04 1.25 -0.71 0.05 17.72 1.19 -0.72 0.05

^a^All slopes are significant, p<0.001

*Moore-1: recommended equation, age and height [13]; Mirwald: original equation [3]
