# Supplementary material for: Modified Maturity Offset Prediction Equations: Validation in Independent Longitudinal Samples of Boys and Girls
Source: Sports Med. 2017 Jun 12;48(1):221–36. doi: 10.1007/s40279-017-0750-y (PMC5752743; doi:10.1007/s40279-017-0750-y)
Supplement: Supplementary file 2 — Supplementary Table 2A Intercepts and slopes of Bland–Altman regressions of the difference between predicted minus observed ages at peak height velocity (PHV) [y-axis] on the mean of predicted and observed ages at PHV (x-axis) for the three prediction equations in boys by years before and after observed age at PHV. Supplementary Table 2B Intercepts and slopes of Bland–Altman regressions of the difference between predicted minus observed ages at peak height velocity (PHV) [y-axis] on the mean of predicted and observed ages at PHV (x-axis) for the two prediction equations in girls three years before to 3 years after observed age at PHV (DOCX 17 kb) [file 40279_2017_750_MOESM2_ESM.docx]

Supplementary Table 2A. Intercepts and slopes of the Bland-Altman regressions of the difference between predicted age at PHV minus observed age at PHV (*y*-axis) and the mean of predicted and observed ages at PHV (*x*-axis) for the three prediction equations* in boys by years before and after observed age at PHV.

Years Moore-1 Moore-2 Mirwald

re PHV N Intercept SE Slope SE Intercept SE Slope SE Intercept SE Slope SE

-3 177 8.93 0.45 -0.69 0.04^a^ 10.76 0.45 -0.83 0.03 7.78 0.54 -0.58 0.04

-2 187 9.53 0.50 -0.86 0.04 11.70 0.54 -0.86 0.04 8.18 0.60 -0.58 0.04

-1 188 10.27 0.50 -0.72 0.04 12.13 0.55 -0.88 0.04 8.75 0.58 -0.59 0.04

0 179 10.08 0.61 -0.71 0.04 12.02 0.67 -0.86 0.05 8.22 0.71 -0.56 0.05

1 184 9.66 0.65 -0.68 0.05 11.92 0.70 -0.86 0.05 7.49 0.74 -0.51 0.05

2 180 9.38 0.71 -0.68 0.05 12.00 0.76 -0.85 0.05 7.05 0.79 -0.47 0.06

3 185 9.40 0.79 -0.64 0.06 12.23 0.89 -0.84 0.06 7.03 0.88 -0.45 0.06

^a^All slopes are significant, p<0.001

*Moore-1: recommended equation, age and sitting height, Moore-2: alternative equation, age and height [13], Mirwald: original equation [3]

Supplementary Table 2B. Intercepts and slopes of the Bland-Altman regressions of the difference between predicted age at PHV minus observed age at PHV (*y*-axis) on the mean of predicted and observed ages at PHV (*x*-axis) for the two prediction equations* in girls three years before to three years after observed age at PHV.

Years Moore Mirwald

re PHV N Intercept SE Slope SE Intercept SE Slope SE

-3 176 9.80 0.45 -0.86 0.04^a^ 7.55 0.56 -0.72 0.05

-2 181 10.50 0.43 -0.87 0.04 8.11 0.51 -0.76 0.05

-1 187 11.16 0.48 -0.86 0.04 8.35 0.53 -0.74 0.05

0 188 11.36 0.54 -0.83 0.04 8.10 0.54 -0.72 0.05

1 187 11.96 0.62 -0.81 0.04 8.56 0.57 -0.72 0.05

2 196 11.25 0.69 -0.75 0.05 8.04 0.58 -0.67 0.05

3 181 11.41 0.77 -0.72 0.05 8.22 0.65 -0.64 0.06

^a^All slopes are significant, p<0.001

*Moore-1: recommended equation, age and height [13]; Mirwald: original equation [3]
