# Supplementary material for: Modified Maturity Offset Prediction Equations: Validation in Independent Longitudinal Samples of Boys and Girls
Source: Sports Med. 2017 Jun 12;48(1):221–36. doi: 10.1007/s40279-017-0750-y (PMC5752743; doi:10.1007/s40279-017-0750-y)
Supplement: Supplementary file 3 — Supplementary Table 3A Descriptive statistics for actual maturity offset, predicted maturity offset and ages at peak height velocity (PHV), and the difference of predicted minus observed age at PHV with the three prediction equations in early, average and late maturing boys by age group. Supplementary Table 3B Descriptive statistics for actual maturity offset, predicted maturity offset and ages at peak height velocity (PHV), and the difference of predicted minus observed age at PHV with the two equations in early, average and late maturing girls by age group (DOCX 30 kb) [file 40279_2017_750_MOESM3_ESM.docx]

Supplementary Table 3A. Descriptive statistics for actual maturity offset, predicted maturity offset and ages at PHV, and the difference of predicted minus observed age at PHV with the three prediction equations* in early, average and late maturing boys by age group

Maturity Offset, yrs Predicted Age at PHV minus

Predicted Predicted Age at PHV, yrs Observed Age at PHV, yrs

Age, Actual Moore-1 Moore-2 Mirwald Moore-1 Moore-2 Mirwald Moore-1 Moore-2 Mirwald

yrs N M SD M SD M SD M SD M SD M SD M SD M SD M SD M SD

EARLY

8 32 -4.45 0.50 -4.20 0.24 -4.27 0.22 -4.44 0.32 12.31 0.20 12.38 0.19 12.54 0.30 -0.25 0.45 -0.18 0.45 -0.01 0.50

9 34 -3.53 0.49 -3.64 0.32 -3.67 0.31 -3.88 0.41 12.67 0.21 12.70 0.19 12.91 0.31 0.11 0.42 0.13 0.43 0.35 0.47

10 35 -2.52 0.49 -2.96 0.33 -2.99 0.32 -3.20 0.42 13.00 0.23 13.03 0.22 13.25 0.34 0.44 0.42^c^ 0.47 0.42^c^ 0.68 0.47^c^

11 36 -1.54 0.48 -2.27 0.33 -2.30 0.34 -2.52 0.43 13.31 0.25 13.33 0.23 13.55 0.37 0.74 0.41^c^ 0.76 0.41^c^ 0.98 0.46^c^

12 36 -0.58 0.48 -1.56 0.42 -1.50 0.43 -1.79 0.52 13.55 0.27 13.49 0.27 13.78 0.40 0.98 0.38^c^ 0.92 0.39^c^ 1.21 0.44^c^

13 35 0.40 0.49 -0.57 0.49 -0.51 0.48 -0.73 0.60 13.55 0.31 13.49 0.30 13.71 0.43 0.97 0.40^c^ 0.91 0.41^c^ 1.13 0.46^c^

14 36 1.41 0.48 0.42 0.48 0.42 0.44 0.30 0.59 13.56 0.33 13.56 0.30 13.67 0.47 0.99 0.45^c^ 0.99 0.47^c^ 1.10 0.53^c^

15 35 2.44 0.43 1.31 0.45 1.21 0.44 1.17 0.56 13.70 0.35 13.80 0.31 13.84 0.49 1.14 0.49^c^ 1.23 0.49^c^ 1.27 0.57^c^

16 35 3.45 0.50 2.06 0.46 1.90 0.46 1.85 0.55 13.97 0.35 14.13 0.32 14.17 0.47 1.40 0.53^c^ 1.55 0.54^c^ 1.60 0.61^c^

17 32 4.47 0.57 2.78 0.49 2.58 0.47 2.49 0.58 14.26 0.39 14.47 0.34 14.55 0.51 1.68 0.57^c^ 1.89 0.57^c^ 1.97 0.65^c^

18 29 5.49 0.51 3.49 0.50 3.26 0.49 3.10 0.60 14.58 0.41 14.81 0.36 14.96 0.54 2.00 0.56^c^ 2.23 0.55^c^ 2.39 0.65^c^

AVERAGE

8 115 -5.83 0.58 -4.22 0.21 -4.30 0.20 -4.48 0.27 12.35 0.18 12.43 0.18 12.61 0.24 -1.61 0.52^c^ -1.54 0.53^c^ -1.35 0.53^c^

9 113 -4.92 0.60 -3.66 0.26 -3.74 0.26 -3.93 0.32 12.70 0.19 12.77 0.19 12.96 0.26 -1.26 0.52^c^ -1.18 0.52^c^ -0.99 0.52^c^

10 110 -3.88 0.58 -2.98 0.27 -3.03 0.27 -3.26 0.33 13.05 0.20 13.11 0.21 13.33 0.27 -0.91 0.50^c^ -0.86 0.51^c^ -0.63 0.51^c^

11 113 -2.92 0.57 -2.33 0.27 -2.38 0.29 -2.63 0.34 13.40 0.22 13.45 0.23 13.69 0.31 -0.58 0.52^c^ -0.53 0.52^c^ -0.28 0.54^c^

12 110 -1.95 0.58 -1.74 0.30 -1.71 0.33 -2.07 0.38 13.75 0.24 13.72 0.25 14.09 0.33 -0.22 0.51^a^ -0.25 0.52^b^ 0.11 0.53

13 111 -0.97 0.58 -0.95 0.38 -0.86 0.38 -1.26 0.46 13.96 0.28 13.87 0.30 14.27 0.38 -0.02 0.45 -0.10 0.47 0.30 0.46^c^

14 115 0.06 0.57 0.01 0.46 0.09 0.45 -0.27 0.58 14.01 0.37 13.92 0.36 14.28 0.50 0.05 0.41 -0.04 0.44 0.32 0.44^c^

15 112 1.04 0.57 1.03 0.48 1.06 0.45 0.80 0.60 13.97 0.40 13.94 0.38 14.21 0.53 0.02 0.44 -0.01 0.49 0.25 0.48^c^

16 112 2.00 0.57 1.92 0.46 1.87 0.44 1.68 0.56 14.06 0.38 14.11 0.37 14.29 0.50 0.08 0.51 0.13 0.57^b^ 0.32 0.55^c^

17 110 3.01 0.58 2.73 0.45 2.60 0.43 2.45 0.54 14.25 0.39 14.38 0.37 14.53 0.49 0.28 0.55^c^ 0.41 0.60^c^ 0.56 0.61^c^

18 106 4.00 0.60 3.48 0.44 3.30 0.45 3.12 0.51 14.49 0.38 14.67 0.40 14.86 0.47 0.51 0.59^c^ 0.69 0.65^c^ 0.88 0.63^c^

LATE

8 39 -7.62 0.62 -4.30 0.26 -4.40 0.25 -4.59 0.33 12.37 0.18 12.47 0.19 12.67 0.26 -3.32 0.60^c^ -3.22 0.60^c^ -3.02 0.63^c^

9 39 -6.73 0.62 -3.78 0.30 -3.85 0.29 -4.08 0.37 12.74 0.22 12.82 0.21 13.05 0.30 -2.95 0.59^c^ -2.88 0.59^c^ -2.64 0.63^c^

10 40 -5.67 0.61 -3.11 0.31 -3.17 0.31 -3.44 0.38 13.12 0.24 13.18 0.23 13.45 0.32 -2.56 0.60^c^ -2.50 0.60^c^ -2.23 0.64^c^

11 37 -4.66 0.53 -2.49 0.31 -2.56 0.30 -2.84 0.39 13.47 0.27 13.54 0.25 13.82 0.37 -2.17 0.55^c^ -2.11 0.56^c^ -1.82 0.60^c^

12 40 -3.75 0.60 -1.91 0.34 -1.90 0.36 -2.31 0.42 13.85 0.27 13.84 0.27 14.24 0.36 -1.83 0.60^c^ -1.84 0.61^c^ -1.44 0.64^c^

13 37 -2.79 0.62 -1.27 0.39 -1.18 0.39 -1.70 0.46 14.18 0.30 14.09 0.30 14.62 0.39 -1.52 0.60^c^ -1.61 0.62^c^ -1.09 0.63^c^

14 40 -1.76 0.60 -0.55 0.42 -0.43 0.42 -1.02 0.50 14.47 0.34 14.36 0.34 14.95 0.44 -1.21 0.60^c^ -1.33 0.62^c^ -0.74 0.65^c^

15 37 -0.74 0.58 0.23 0.49 0.40 0.51 -0.26 0.58 14.67 0.39 14.50 0.40 15.16 0.50 -0.97 0.50^c^ -1.14 0.55^c^ -0.48 0.54^b^

16 38 0.21 0.59 1.23 0.53 1.41 0.51 0.76 0.65 14.68 0.47 14.50 0.44 15.15 0.61 -1.02 0.52^c^ -1.20 0.55^c^ -0.55 0.57^c^

17 37 1.23 0.61 2.28 0.60 2.37 0.56 1.85 0.72 14.63 0.52 14.53 0.47 15.06 0.65 -1.05 0.57^c^ -1.14 0.60^c^ -0.62 0.63^c^

18 39 2.21 0.61 3.15 0.61 3.19 0.55 2.68 0.71 14.74 0.53 14.71 0.48 15.22 0.64 -0.94 0.65^c^ -0.98 0.70^c^ -0.46 0.71^c^

^a^p<0.05, ^b^p<0.01, ^c^p<0.001

*Moore-1: recommended equation, age and sitting height, Moore-2: alternative equation, age and height [13], Mirwald: original equation [3]

Supplementary Table 3B. Descriptive statistics for actual maturity offset, predicted maturity offset and ages at PHV, and the difference of predicted age at PHV minus observed age at PHV (criterion) with the two equations* in early, average and late maturing girls by age group

Maturity Offset, yrs Difference, Predicted Minus

Predicted Predicted Age at PHV, yrs Actual Age at PHV, yrs

Age, Actual Moore-1 Mirwald Moore-1 Mirwald Moore-1 Mirwald

yrs N M SD M SD M SD M SD M SD M SD M SD

EARLY

8 28 -2.20 0.58 -3.45 0.28 -3.38 0.41 11.44 0.19 11.37 0.31 1.25^c^ 0.48 1.18^c^ 0.50

9 23 -1.19 0.58 -2.72 0.36 -2.64 0.48 11.68 0.22 11.60 0.35 1.53^c^ 0.47 1.46^c^ 0.51

10 27 -0.24 0.60 -1.87 0.43 -1.76 0.52 11.83 0.25 11.72 0.35 1.64^c^ 0.45 1.54^c^ 0.49

11 27 0.72 0.60 -1.00 0.40 -0.89 0.50 11.91 0.23 11.80 0.34 1.72^c^ 0.47 1.61^c^ 0.50

12 24 1.83 0.55 0.07 0.41 0.17 0.49 11.97 0.27 11.87 0.34 1.76^c^ 0.51 1.66^c^ 0.55

13 26 2.79 0.56 0.79 0.43 0.82 0.50 12.17 0.30 12.14 0.36 2.00^c^ 0.54 1.97^c^ 0.59

14 27 3.78 0.55 1.55 0.44 1.46 0.45 12.42 0.32 12.50 0.34 2.23^c^ 0.55 2.31^c^ 0.58

15 27 4.85 0.53 2.32 0.44 2.14 0.43 12.72 0.36 12.90 0.37 2.53^c^ 0.58 2.71^c^ 0.60

16 25 5.82 0.53 3.00 0.44 2.65 0.38 13.03 0.40^a^ 13.36 0.40 2.82^c^ 0.58 3.15^c^ 0.61

AVERAGE

8 138 -3.87 0.62 -3.51 0.26 -3.56 0.34 11.56 0.17 11.61 0.25 -0.35^c^ 0.52 -0.30^c^ 0.50

9 125 -2.89 0.60 -2.83 0.30 -2.87 0.36 11.81 0.21 11.86 0.29 -0.06 0.52 -0.02 0.51

10 132 -1.90 0.61 -2.07 0.34 -2.14 0.41 12.08 0.24 12.16 0.32 0.17 0.51 0.24^b^ 0.49

11 130 -0.87 0.59 -1.20 0.36 -1.28 0.42 12.24 0.28 12.32 0.36 0.33^c^ 0.48 0.41^c^ 0.46

12 131 0.13 0.56 -0.26 0.41 -0.34 0.47 12.29 0.33 12.39 0.42 0.39^c^ 0.46 0.47^c^ 0.43

13 134 1.09 0.58 0.69 0.44 0.56 0.47 12.32 0.34 12.45 0.39 0.40^c^ 0.51 0.53^c^ 0.46

14 140 2.10 0.57 1.56 0.43 1.37 0.44 12.46 0.36 12.64 0.39 0.54^c^ 0.58 0.73^c^ 0.54

15 130 3.11 0.58 2.35 0.44 2.08 0.42 12.66 0.39 12.93 0.39 0.76^c^ 0.64 1.02^c^ 0.60

16 123 4.08 0.58 3.06 0.44 2.68 0.41 12.94 0.41 13.31 0.40 1.01^c^ 0.66 1.38^c^ 0.61

LATE

8 30 -5.31 0.50 -3.62 0.21 -3.71 0.29 11.69 0.19 11.78 0.29 -1.69^c^ 0.44 -1.60^c^ 0.49

9 27 -4.43 0.52 -3.02 0.24 -3.10 0.33 11.96 0.21 12.04 0.31 -1.41^c^ 0.46 -1.33^c^ 0.51

10 29 -3.38 0.51 -2.30 0.26 -2.42 0.34 12.27 0.22 12.39 0.33 -1.09^c^ 0.43 -0.96^c^ 0.48

11 28 -2.41 0.52 -1.54 0.29 -1.66 0.39 12.53 0.24 12.65 0.37 -0.87^c^ 0.46 -0.75^c^ 0.53

12 26 -1.32 0.56 -0.71 0.36 -0.90 0.39 12.74 0.23 12.93 0.29 -0.61^c^ 0.42 -0.42^b^ 0.45

13 30 -0.36 0.56 0.21 0.39 -0.03 0.43 12.81 0.30 13.05 0.37 -0.57^c^ 0.41 -0.33 0.45

14 29 0.64 0.56 1.22 0.42 0.94 0.46 12.80 0.30 13.07 0.37 -0.58^c^ 0.40 -0.31 0.43

15 29 1.61 0.56 2.10 0.44 1.78 0.45 12.89 0.33 13.23 0.36 -0.49^c^ 0.43 -0.15 0.43

16 25 2.61 0.55 2.92 0.42 2.47 0.37 13.10 0.39 13.54 0.39 -0.31 0.53 0.13 0.51

^a^p<0.05, ^b^p<0.01, ^c^p<0.001

**Moore-1: recommended equation, age and height [13]; Mirwald: original equation [3]
