# Supplementary material for: Modified Maturity Offset Prediction Equations: Validation in Independent Longitudinal Samples of Boys and Girls
Source: Sports Med. 2017 Jun 12;48(1):221–36. doi: 10.1007/s40279-017-0750-y (PMC5752743; doi:10.1007/s40279-017-0750-y)
Supplement: Supplementary file 4 — Supplementary Table 4A Descriptive statistics for predicted maturity offset and ages at peak height velocity (PHV), and the difference of predicted age at PHV minus observed age at PHV for the three equations in early, average and late maturing boys from −3 to +3 years of observed age at PHV. Supplementary Table 4B Descriptive statistics for predicted maturity offset and ages at peak height velocity (PHV), and the difference of predicted age at PHV minus observed age at PHV for the two equations in early, average and late maturing girls from −3 to +3 years of observed age at PHV (DOCX 23 kb) [file 40279_2017_750_MOESM4_ESM.docx]

Supplementary Table 4A. Descriptive statistics for predicted maturity offset and ages at PHV, and the difference of predicted age at PHV minus observed age at PHV for the three equations* in early, average and late maturing boys from -3 to +3 years of observed age at PHV

Predicted Predicted Age at PHV Minus

Maturity Offset, yrs Predicted Age at PHV, yrs Observed Age at PHV, yrs

Years Moore-1 Moore-2 Mirwald Moore-1 Moore-2 Mirwald Moore-1 Moore-2 Mirwald

re PHV N M SD M SD M SD M SD M SD M SD M SD M SD M SD

EARLY

-3 35 -3.31 0.38 -3.34 0.39 -3.55 0.45 12.83 0.28 12.87 0.26 13.08 0.37 0.27 0.31 0.30 0.33^a^ 0.51 0.38^c^

-2 35 -2.58 0.45 -2.61 0.44 -2.82 0.52 13.18 0.31 13.21 0.29 13.42 0.42 0.61 0.31^c^ 0.64 0.30^c^ 0.85 0.39^c^

-1 36 -1.84 0.49 -1.82 0.54 -2.08 0.56 13.48 0.32 13.46 0.28 13.73 0.43 0.91 0.30^c^ 0.89 0.33^c^ 1.16 0.39^c^

0 33 -0.96 0.60 -0.90 0.61 -1.15 0.68 13.54 0.29 13.48 0.29 13.73 0.41 1.00 0.39^c^ 0.93 0.39^c^ 1.19 0.47^c^

1 34 0.03 0.59 0.06 0.58 -0.09 0.67 13.52 0.33 13.49 0.31 13.64 0.46 0.96 0.40^c^ 0.93 0.40^c^ 1.08 0.49^c^

2 37 0.95 0.59 0.90 0.56 0.83 0.66 13.63 0.36 13.68 0.32 13.75 0.49 1.05 0.40^c^ 1.10 0.39^c^ 1.17 0.49^c^

3 34 1.73 0.61 1.59 0.57 1.57 0.67 13.81 0.37 13.95 0.35 13.97 0.50 1.26 0.43^c^ 1.40 0.41^c^ 1.42 0.52^c^

AVERAGE

-3 112 -2.38 0.38 -2.42 0.42 -2.67 0.41 13.37 0.33 13.42 0.30 13.67 0.42 -0.61 0.36^c^ -0.56 0.38^c^ -0.31 0.40^c^

-2 109 -1.77 0.40 -1.74 0.47 -2.10 0.43 13.75 0.33 13.71 0.31 14.08 0.42 -0.22 0.39^a^ -0.26 0.43^b^ 0.10 0.42

-1 111 -1.02 0.46 -0.94 0.51 -1.35 0.48 13.97 0.31 13.88 0.32 14.30 0.40 0.01 0.38 -0.07 0.41 0.34 0.41^c^

0 110 -0.07 0.50 0.02 0.54 -0.36 0.55 14.03 0.38 13.93 0.37 14.32 0.50 0.06 0.42 -0.03 0.45 0.35 0.47^c^

1 116 0.98 0.52 1.02 0.55 0.75 0.56 13.97 0.40 13.93 0.39 14.19 0.52 0.00 0.44 -0.04 0.46 0.23 0.49^a^

2 113 1.91 0.49 1.86 0.52 1.69 0.54 14.03 0.42 14.08 0.40 14.25 0.55 0.08 0.45 0.13 0.48 0.30 0.51^c^

3 110 2.75 0.52 2.62 0.56 2.47 0.55 14.22 0.43 14.36 0.41 14.51 0.54 0.24 0.50^b^ 0.38 0.52^c^ 0.53 0.55^c^

LATE

-3 38 -1.40 0.51 -1.33 0.55 -1.84 0.53 14.14 0.37 14.06 0.35 14.58 0.46 -1.55 0.47^c^ -1.62 0.51^c^ -1.11 0.50^c^

-2 37 -0.72 0.46 -0.61 0.50 -1.18 0.51 14.40 0.40 14.29 0.36 14.86 0.51 -1.22 0.41^c^ -1.33 0.46^c^ -0.76 0.47^c^

-1 38 0.05 0.55 0.21 0.60 -0.46 0.58 14.68 0.40 14.52 0.40 15.18 0.51 -1.00 0.50^c^ -1.16 0.55^c^ -0.50 0.53^b^

0 36 1.03 0.62 1.20 0.63 0.56 0.68 14.68 0.45 14.50 0.43 15.14 0.58 -1.01 0.52^c^ -1.19 0.55^c^ -0.55 0.57^b^

1 38 2.07 0.72 2.20 0.70 1.62 0.78 14.63 0.50 14.49 0.46 15.07 0.63 -1.08 0.59^c^ -1.22 0.59^c^ -0.64 0.65^c^

2 37 3.01 0.68 2.06 0.67 2.54 0.74 14.69 0.55 14.64 0.50 15.15 0.67 -0.97 0.63^c^ -1.02 0.65^c^ -0.51 0.69^b^

3 33 3.70 0.59 3.70 0.65 3.19 0.66 14.85 0.55 14.85 0.53 15.36 0.66 -0.70 0.56^c^ -0.70 0.64^c^ -0.19 0.63

^a^p<0.05, ^b^p<0.01, ^c^p<0.001

*Moore-1: recommended equation, age and sitting height, Moore-2: alternative equation, age and height [13], Mirwald: original equation [3]

Supplementary Table 4B. Descriptive statistics for predicted maturity offset and ages at PHV, and the difference of predicted age at PHV minus observed age at PHV for the two equations* in early, average and late maturing girls from -3 to +3 years of observed age at PHV

Predicted Age at PHV Minus

Years Maturity Offset, yrs Predicted Age at PHV, yrs Observed Age at PHV, yrs

Relative Moore-1 Mirwald Moore-1 Mirwald Moore-1 Mirwald

to PHV N M SD M SD M SD M SD M SD M SD

EARLY

-3 13 -3.62 0.21 -3.57 0.28 11.36 0.18 11.31 0.27 0.84^c^ 0.17 0.78^c^ 0.24

-2 25 -3.25 0.37 -3.19 0.47 11.52 0.21 11.46 0.32 1.25^c^ 0.33 1.19^c^ 0.41

-1 27 -2.47 0.48 -2.39 0.53 11.73 0.29 11.64 0.41 1.54^c^ 0.39 1.46^c^ 0.45

0 26 -1.58 0.55 -1.46 0.58 11.80 0.26 11.68 0.35 1.63^c^ 0.42 1.51^c^ 0.46

1 26 -0.71 0.52 -0.59 0.55 11.92 0.26 11.79 0.35 1.74^c^ 0.45 1.62^c^ 0.48

2 26 0.22 0.52 0.31 0.54 12.02 0.33 11.93 0.40 1.80^c^ 0.46 1.71^c^ 0.48

3 27 0.97 0.53 1.00 0.54 12.24 0.37 12.21 0.43 2.03^c^ 0.47 2.04^c^ 0.49

AVERAGE

-3 134 -2.87 0.44 -2.92 0.43 11.83 0.29 11.89 0.38 -0.09 0.38 -0.03 0.38

-2 129 -2.11 0.47 -2.17 0.47 12.06 0.30 12.14 0.39 0.15 0.40 0.22^a^ 0.40

-1 131 -1.29 0.50 -1.37 0.49 12.24 0.31 12.33 0.40 0.33^c^ 0.43 0.41^c^ 0.41

0 133 -0.35 0.55 -0.44 0.53 12.30 0.33 12.40 0.43 0.39^c^ 0.46 0.49^c^ 0.43

1 132 0.64 0.58 0.51 0.52 12.30 0.35 12.43 0.42 0.38^c^ 0.48 0.50^c^ 0.43

2 140 1.52 0.57 1.35 0.50 12.43 0.38^a^ 12.60 0.45 0.52^c^ 0.51 0.69^c^ 0.45

3 133 2.30 0.57 2.04 0.49 12.65 0.40 12.90 0.44 0.74^c^ 0.53 1.00^c^ 0.46

LATE

-3 29 -1.98 0.39 -2.10 0.47 12.39 0.27 12.50 0.36 -1.00^c^ 0.36 -0.88^c^ 0.44

-2 27 -1.23 0.38 -1.39 0.39 12.63 0.24 12.79 0.33 -0.72^c^ 0.34 -0.56^c^ 0.35

-1 29 -0.32 0.42 -0.53 0.44 12.80 0.30 13.01 0.39 -0.59^c^ 0.38 -0.38^a^ 0.42

0 29 0.63 0.46 0.36 0.47 12.83 0.32 13.09 0.39 -0.57^c^ 0.40 -0.30 0.42

1 29 1.61 0.46 1.30 0.46 12.82 0.33 13.13 0.39 -0.56^c^ 0.41 -0.26 0.41

2 30 2.47 0.46 2.10 0.42 12.95 0.37 13.31 0.39 -0.44^c^ 0.43 -0.07 0.39

3 21 3.18 0.46 2.69 0.41 13.16 0.38 13.61 0.37 -0.16 0.45 0.33 0.41

^a^p<0.05, ^b^p<0.01, ^c^p<0.001

*Moore-1: recommended equation, age and height [13], Mirwald: original equation [3]
