# Supplementary material for: Modified Maturity Offset Prediction Equations: Validation in Independent Longitudinal Samples of Boys and Girls
Source: Sports Med. 2017 Jun 12;48(1):221–36. doi: 10.1007/s40279-017-0750-y (PMC5752743; doi:10.1007/s40279-017-0750-y)
Supplement: Supplementary file 5 — Supplementary Table 5 Sample sizes and means and standard deviations for the ratio of leg length to sitting height (%) by years before and after observed peak height velocity (PHV) in boys and girls from the Wroclaw Growth Study (WGS) and from the Pediatric Bone Mineral Accrual Study (PBMAS) (DOCX 14 kb) [file 40279_2017_750_MOESM5_ESM.docx]

Supplementary Table 5. Sample sizes, means and standard deviations for the ratio of leg length to sitting height (%) by years before and after observed PHV in boys and girls from the Wroclaw Growth Study (WGS) and from the Pediatric Bone Mineral Accrual Study (PBMAS) [3].

Boys Girls

Years WGS PBMAS WGS PBMAS

PHV n Mean SD n Mean SD n Mean SD n Mean SD

-3 189 89.5 4.6 68 89.6 3.4 176 86.7 4.4 46 89.1 3.9

-2 181 91.8 4.8 98 91.1 3.4 182 88.1 4.1 73 90.3 3.8

-1 184 93.5 4.4 125 92.5 3.9 186 89.6 4.1 96 91.2 3.7

0 179 94.2 4.3 141 93.2 4.0 190 90.7 4.5 124 91.4 3.9

+1 188 93.4 4.4 110 92.3 4.2 188 90.7 4.3 121 90.3 3.5

+2 187 91.8 4.6 68 90.4 3.9 196 89.2 4.3 97 89.3 4.2

+3 177 90.6 4.5 25 89.6 3.0 180 88.0 4.5 33 88.5 3.9
